# Sparse Representation Based Frequency Detection and Uncertainty Reduction in Blade Tip Timing Measurement for Multi-Mode Blade Vibration Monitoring

**DOI:** 10.3390/s17081745

**Published:** 2017-07-30

**Authors:** Minghao Pan, Yongmin Yang, Fengjiao Guan, Haifeng Hu, Hailong Xu

**Affiliations:** Science and Technology on Integrated Logistics Support Laboratory, National University of Defense Technology, Changsha 410073, China; panminghao15@nudt.edu.cn (M.P.); guanfengjiao@nudt.edu.cn (F.G.); hhf_online@163.com (H.H.); xhlym1@163.com (H.X.)

**Keywords:** blade tip timing, measurement uncertainty, vibration frequency spectrum recovery, sparse representation, multi-mode vibration

## Abstract

The accurate monitoring of blade vibration under operating conditions is essential in turbo-machinery testing. Blade tip timing (BTT) is a promising non-contact technique for the measurement of blade vibrations. However, the BTT sampling data are inherently under-sampled and contaminated with several measurement uncertainties. How to recover frequency spectra of blade vibrations though processing these under-sampled biased signals is a bottleneck problem. A novel method of BTT signal processing for alleviating measurement uncertainties in recovery of multi-mode blade vibration frequency spectrum is proposed in this paper. The method can be divided into four phases. First, a single measurement vector model is built by exploiting that the blade vibration signals are sparse in frequency spectra. Secondly, the uniqueness of the nonnegative sparse solution is studied to achieve the vibration frequency spectrum. Thirdly, typical sources of BTT measurement uncertainties are quantitatively analyzed. Finally, an improved vibration frequency spectra recovery method is proposed to get a guaranteed level of sparse solution when measurement results are biased. Simulations and experiments are performed to prove the feasibility of the proposed method. The most outstanding advantage is that this method can prevent the recovered multi-mode vibration spectra from being affected by BTT measurement uncertainties without increasing the probe number.

## 1. Introduction

High-speed rotor blades in turbines and compressors are often subject to several sources of excitations that lead to various forced vibration responses. These vibrations are common causes of blade damages that may even result in catastrophic failures. As a non-contacting measurement technology, BTT [1] can be used to identify vibration problems in rotor blades through recording the arrival time of the blades at each probe installed in the casing. However, the sequence of blade tip deflections computed by BTT signals is inherently under-sampled [2], which makes the detection of blade vibration frequencies much harder.

Many methods have been proposed for detecting vibration frequencies from the BTT data. Carrington [3] compared two global autoregressive-based vibration analysis methods. Salhi [4] used an interpolation technique to reconstruct under-sampled BTT signals. However, all of the above techniques only adapted to the case that each blade responds at a single harmonic frequency. Studies [5,6,7] have proved that multiple frequencies appear in the frequency response of blade displacement when the blade is cracked or mistuned. Gallego-Garrido [8] proposed a class of auto-based methods to process BTT data from blades undergoing two simultaneous resonances. Kharyton [9] proposed a method for identifying multi-modes using FFT-like algorithms together with non-uniform Fourier transform. Lin [10] proposed a novel BTT analysis method based on compressive sensing theorem for reconstructing unknown multi-mode blade vibration signals. However, relatively more probes are required with the increase of responding modes in those methods. In addition, the influence of measurement uncertainties is not considered in the data processing. Thus, the proposed algorithms are not guaranteed to get the reliable vibration frequencies under the impact of measurement errors.

Several factors that contribute to uncertainties in BTT measurement have been reported. Satish [11] provided an insight into many probable sources of BTT measurement errors in both engine systems and measurement systems using analytical and experimental techniques. Guo [12] performed a numerical analysis on the rising speed of tip-timing signals and indicated that a variable gain amplifier could minimize the tip-timing error. Berdanier [13] and Lawson [14] analyzed uncertainties on the capacitance probe measurement system and proposed two main sources due to analogue to digital (A/D) conversion processes and signal noise. However, those analyses lack quantitative expressions of the relationship between those mentioned uncertainties and blade vibration measurement results. Hu [15] estimated the reconstruction error affected by parameters in interpolating function respectively. However, approaches for removing uncertainties in those parameters was not proposed.

To overcome the limitations stated above, a novel approach based on the sparse representation theorem for multi-mode vibration frequency detection is presented with the correspondent uncertainty reduction method. Sparse representation (SR) and compressed sensing (CS) have been widely applied to the recovery of signals with a sparse nature [16,17] from under-sampling data accurately. Compared with other predominant techniques, the proposed method has two main advantages: firstly, multiple harmonic frequencies can be extracted from under-sampled BTT data collected by no more than four probes. Experiments have confirmed that the upper boundary of the harmonic frequency number that can be detected simultaneously is no less than four. Secondly, the sensitivity of recovered frequency spectrum to BTT measurement uncertainty can be reduced.

This paper is organized as follows: a BTT sampling mathematical model is built in Section 2. In Section 3, recovery of multi-mode vibration frequency spectra by the theory of sparse representation is introduced. Then the improved frequency spectra recovery method is adapted to the case of biased BTT data in Section 4. In Section 5 and Section 6, simulation and experimental results are presented to evaluate the feasibility of proposed methods. Finally, conclusions are reported in Section 7.

## 2. Mathematical Model of the Blade Tip Timing Sampling

### 2.1. Formulations of Blade Tip Deflections

Without attaching to the blades, the blade tip deflections will be determined from the time of arrival (TOA) of each tip relative to the arrival time of a rotating reference point in BTT measurement.

**Figure 1:**
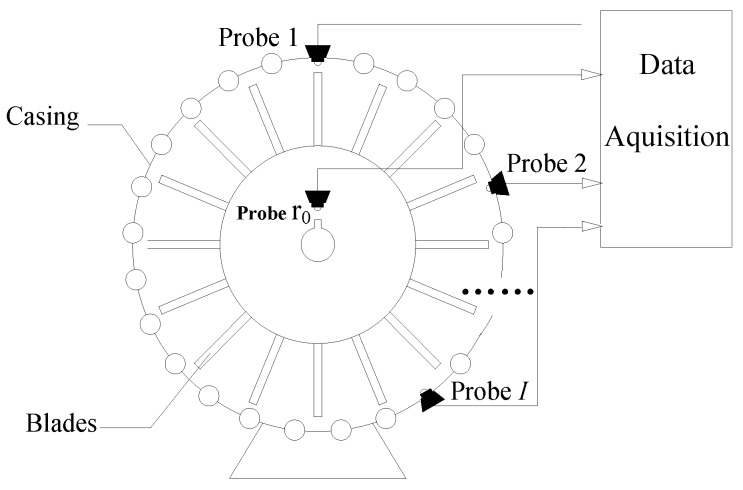
Blade tip timing system.

As shown in Figure 1, *I* probes are mounted circumferentially around a rotor with *K* blades so that the times when the blades pass the probes can be measured. The locations of the *i*th probe and the *k*th blade can be described as angles $\alpha_{i}$ and $\theta_{k}$, respectively. The other probe $r_{0}$ is mounted above the shaft to collect once per revolution signals as reference signals.

Assuming that the blade disk rotates clockwise, when a non-vibrating blade *k* passes the *i*th probe at the *n*th rotation, the expected TOA $t_{\mathrm{expected}}^{i,n}$ can be calculated [18] as:

(1) $$t_{\mathrm{expected}}^{i,n}=\frac{T_{n}}{2\pi }\left(2\pi n+\alpha_{i}-\theta_{k}\right),$$

where *T_n_* is the constant rotating period. Hence the $t_{\mathrm{expected}}^{i,n}$ can be used to calculate deflection of each blade.

By comparing the detected TOAs to the ideal ones of the blade, the blade tip displacement can be obtained as follows:

(2) $$\Delta t=t_{\mathrm{expected}}-t_{\mathrm{actual}},$$

(3) $$d\left(t_{\mathrm{actual}}\right)=\frac{2\pi R_{\mathrm{tip}}}{T_{n}}\Delta t,$$

where $R_{\mathrm{tip}}$ is the radius from the rotation axis of the engine to the blade tip and $t_{\mathrm{actual}}$ is the actual TOA that the probe records for each blade.

### 2.2. Function of BTT Signals

Consider a continued blade vibration signal *d*(*t*) for an arbitrary blade *k*. The TOA of each blade trigger the probe once in a rotation based on the principle of BTT measurements. Assuming $\theta_{k}=0$ and the rotation period *T_n_* is a constant, the BTT sampling function of an arbitrary blade *k* during *N* rotations can be formulated as follows:

(4) $$y_{I}^{k}(t)=d\left(t\right)\overset{I}{\underset{i=1}{\sum }}\overset{N}{\underset{n=1}{\sum }}\delta \left(t-\left(n-1\right)T_{n}-\frac{\alpha_{i}}{2\pi }T_{n}\right),$$

where δ( ) is the Dirac delta function, *d*(*t*) is the blade tip vibration signal. Therefore, *I* BTT data are sampled in each of *N* periodicities, where data sampled by the *i*th probe is shifted by $\frac{\alpha_{i}}{2\pi }T_{n}$ from the origin one.

As illustrated in Equation (4), the sampling frequency of the BTT measurement is determined by the number of probes and the rotating speed. Unfortunately, the rotating frequency is always much lower than the maximal blade vibration frequency. Therefore, BTT signals are under-sampled in most of the cases due to the bound of probe number.

Generally, the response for a single mode blade vibration is modeled as a harmonic wave, which is classified into ‘Synchronous’ and ‘Asynchronous’ according to the vibration frequency [3]. However, actual blades may yield responses at coupled-modes vibrations. Therefore, the response of multi-mode blade vibration could be expressed as the following formula:

(5) $$d\left(t\right)=\sum_{i=1}^{M}A_{i}\sin\left(2\pi f_{i}t+\varphi_{i}\right),$$

where {*A_i_*} are amplitudes of vibrations, {*f_i_*} are dominant frequencies, {*ϕ_i_*} are phases of the response and *M* is the number of vibration modes. In general, harmonic features in *d*(*t*) are corresponding to different kinds of excitations and fatigues of blades.

## 3. Vibration Frequency Detection Based on Sparse Representation Theory

### 3.1. Sparse Representation Mathematical Model of the BTT Measurement

Suppose there are *L* imaginary probes installed uniformly around the casing as shown in Figure 1. For each rotation, *L* data are sampled from vibration signal. Thus, the sampling rate of this system equals to *Lf_r_* ( *f_r_ = 1/T_n_* ). According to Nyquist sampling theorem [19], if the sampling rate of this system satisfies *Lf_r_*/2 > *f_blade_* these calculated deflection data contain all information about the frequency of vibration signals. Considering the engine constraints, however, only a limited number of probes can be mounted. Therefore, as shown in Figure 1, only *I* probes are embedded in certain angular positions selected from these *L* ones.

The sampling stream for an arbitrary blade vibration signal could be then viewed as discarding all but *I* actual samples in every rotation of *L* imaginary samples periodically. An integer set $\Lambda =\left\{\lambda_{i}|i=1,2,\cdots ,I\right\}$ describes the position of *I* probes. The *i*th probe placement is then written as $\alpha_{i}=2\pi \lambda_{i}/L$. According to Equation (4), for an arbitrary blade, the vibration signals recorded by *I* probes can be represented as a vector of acquired samples y[j]  (1≤j≤IN), where the sampling sequence is under-sampled.

Suppose the vibration signal of an arbitrary blade can be recorded by *L* uniformly displaced probes, then the angular positions of *L* probes can be formulated as 2πl/L  (1≤l≤L) respectively. Based on Equation (4), the imaginary measurement vector can be written as:

(6) $$x\left[\tilde{j}\right]=d\left(t\right)\overset{L}{\underset{l=1}{\sum }}\overset{N}{\underset{n=1}{\sum }}\delta \left[t-\left(n-1\right)T_{n}-\frac{l}{L}T_{n}\right]\;\left(1\leq \tilde{j}\leq LN\right).$$

Obviously, $x\left[\tilde{j}\right]$ includes the frequency information of blade vibration signal, which can be solved through Fourier transform according to the celebrated Shannon sampling theorem.

**Figure 2:**
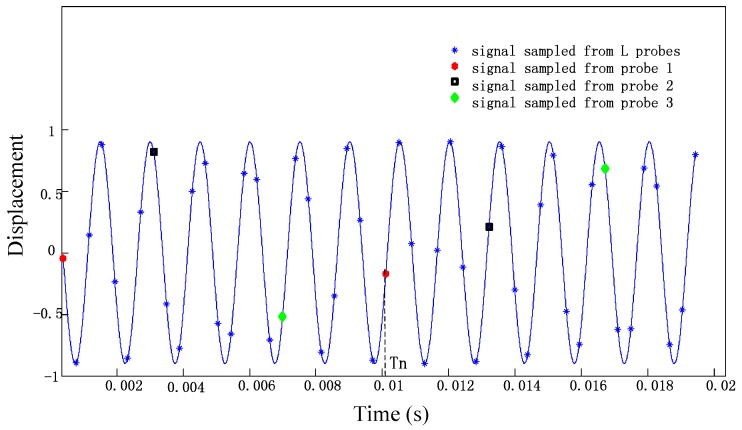
Blade tip displacement data sampled by different sensors in two rotating periods.

Signals sampled in two periods from a vibration waveform in vector y[j] and $x\left[\tilde{j}\right]$ are presented in Figure 2. Therefore, the relationship between y[j] and $x\left[\tilde{j}\right]$ can be converted into a matrix equation.

(7) $$y\left[j\right]=\Phi x\left[\tilde{j}\right],$$

where matrix $\Phi ={\left[\begin{array}{cccccc} 1 & 0 & \cdots & \cdots & \cdots & 0\\ 0 & \cdots & 1 & 0 & \cdots & 0\\ \vdots & \cdots & \cdots & \cdots & \cdots & \vdots \\ 0 & \cdots & \cdots & 1 & \cdots & 0 \end{array}\right]}_{p\times q}$ is determined by the sampling time sequence of *I* probes in each rotation. The number of row *p* and column *q* of the matrix **Φ**, which determined by the scale of y[j] and $x\left[\tilde{j}\right]$, satisfy *p* = *IN* and *q* = *LN* respectively. Matrix **Ф** has entries φ(•,k)=1 in each row. where $\frac{k}{L}T_{n}$ equals to the arriving time of the blade in each rotation, i.e., $k=\lambda_{i}+nL$ (*i* = 1, 2, ..., *I*, *n* = 1,2, ..., *N*).

The frequency of the blade vibration can be derived in the frequency-domain. Hence the Fourier transform of $x\left[\tilde{j}\right]$ can be formulated as follows:

(8) $$\Psi_{FFT}x\left[\tilde{j}\right]=\theta \left(f\right),$$

where $\Psi_{FFT}\in C^{q\times q}$ is the Fourier basis matrix. Apparently, $\theta \left(f\right)\in C^{q}$ is the frequency spectrum of the vibration signal obtained by Fourier transform. As the blade vibration signal has a finite number of modes (harmonic features), θ(f) has a sparse nature. So the non-zero coefficients of vector θ(f) are constrained by number of modes. According to Equation (8), Equation (7) can be transformed into:

(9) $$y\left[j\right]=\Phi \Psi_{FFT}^{T}\theta \left(f\right).$$

Up to now, the sparse representation model of BTT measurement has been built. In addition, the recovery of vibration frequency spectrum θ(f) from Equation (9) has been transformed into a classic Single-Measurement Vector problem [20]. As p<q, Equation (9) is referred to be an overcomplete transform. Thus, further information is needed to uniquely extract the solution of θ(f).

### 3.2. Uniqueness of Solution to SR Model

#### 3.2.1. Restricted Isometry Property and Orthogonalization Preprocessing

The Restricted Isometry Property (RIP) is a useful notion for robust recovery of a sparse signal from an under-sampled measurement vector [21]. As for the single measurement vector model in Equation (9), RIP can be equivalently transformed into the goal that the columns of matrix $\Phi \Psi_{FFT}^{T}$ should be nearly orthogonal [17]. Referring to [22], the following orthogonalization preprocessing procedure can satisfy such property.

Define a sensing matrix **R** as $R=\Phi \Psi_{FFT}^{T}$. The orthogonalization process is done on both sides of Equation (9).

(10) $$QR^{†}y\left[j\right]=QR^{†}R\theta \left(f\right),$$

where **Q** = [orth(**R^T^**)]**^T^**, an orthogonal basis for the range of matrix $R^{T}$, and $R^{†}$ is a pseudoinverse of matrix **R**.

It has been proved [22] that $QR^{†}R=Q$. Therefore, the vibration frequency problem formulated in Equation (7) can be reformulated as:

(11) z=Qθ(f),

where $z=QR^{†}y\left[j\right]$.

Hence the detection of vibration frequency spectrum can be transformed into estimating the solution of θ(f) in Equation (11) with minimal sparsity. It is shown in [22] that the orthogonalized sensing matrix **Q** obeys the RIP. If the length of BTT sampling sequence also obeys the corresponding principles, then the vibration frequency spectrum θ(f) has high probability to be solved from **z** by Basis Pursuit (BP) algorithm that solves the following *l_1_* norm minimization problem [16]:

(12) $$\begin{array}{l} \hat{\theta }\left(f\right)=\arg\;\min{\left\|\theta \left(f\right)\right\|}_{1};\\ s.t.\;z=Q\theta \left(f\right). \end{array}$$

Thus, the frequency of the blade vibration can be extracted from the solution of $\hat{\theta }\left(f\right)$.

#### 3.2.2. Requirement of Probe Number

According to the theory of CS [17], the vibration frequency spectrum can be well recovered from **z** with high probability when the number of validate sampling data $p_{v}$ obeys the following rule:

(13) $$p_{v}=O\left(K_{s}\log\left(q\right)\right),$$

where *K_s_* is the sparsity of θ(f), i.e., ${\left\|\theta \left(f\right)\right\|}_{0}$, *q* is the length of spectrum vector θ(f).

**Figure 3:**
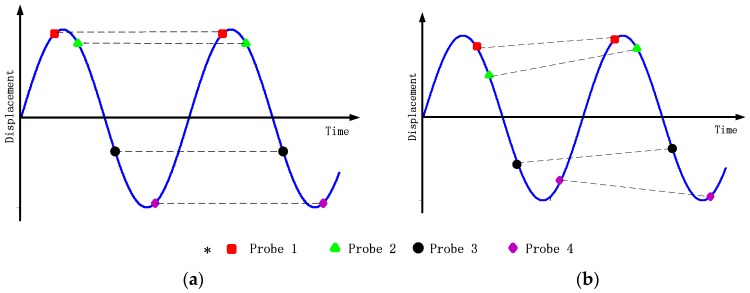
Vibration signal sampled by four optical sensors (**a**) synchronous vibration (**b**) asynchronous vibration.

However, the definition of *p_v_* various from synchronous modes to asynchronous ones. As for synchronous vibration (engine-ordered) [3], whose response frequency is an integer multiple of the rotational speed of the assembly, the same phase of the vibration cycle is detected in each rotation by probes. Thus, the amount of data that is available for the estimation of the response frequency is limited. As shown in Figure 3a, no matter how many measurement results are sampled, the feasible results is equal to the number of probes, i.e., $p_{v}=I$. Asynchronous vibration, however, contains different values of blade displacements in each rotation as shown in Figure 3b, Thus, $p_{v}$ is equal to the length of sampling sequence, i.e., $p_{v}=IN$.

Therefore, the requirement of probe number can be summarized from Equation (13) as follows:

- For the most ideal case, which the blade responds at a synchronous mode, at least four probes are needed to get the unique solution as the length of *q* is in the order of 10^3^ to 10^4^ considering both the computational complexity and the time of engine to rotate in a constant speed.
- For the multi-mode case, which usually mixed with asynchronous resonances caused by flutter [23], three sensors are abundant for the monitoring of multiple mode vibration as long as the sampling time is long enough.

#### 3.2.3. The Coherence of the Sensing Matrix and Choice of Probe Placement Location

According to the theory of SR/CS, the coherence property of matrix **Q** also has effect on the accuracy of the recovery of vibration spectrum θ(f).

The two-sided coherence [24] for matrix **Q** with columns $q_{i}$ is defined as:

(14) $$\mu \left(Q\right)=\underset{i,j;j\neq i}{\max}\frac{\left|\langle q_{i},q_{j}\rangle \right|}{{\left\|q_{i}\right\|}_{2}\cdot {\left\|q_{j}\right\|}_{2}}.$$

For the linear system of equations **z** = **Qθ**(*f*), if a nonnegative solution exists such that ${\left\|\theta \left(f\right)\right\|}_{0}<\frac{1}{2}\left(1+\frac{1}{\mu \left(Q\right)}\right)$, then several efficient algorithms are guaranteed to find it exactly [24]. As θ(f) is the frequency spectrum, the sparsity of spectrum ${\left\|\theta \left(f\right)\right\|}_{0}$ represents the number of modes that contains in vibration signals. To get the high recovery probability of θ(f), μ(Q) must be bounded with an certain value.

According to Equation (7) and Equation (9), **Q** is defined by the number of sensors around the casing and their positions. When the imaginary probe number *L* and probe number *I* are fixed, $\left(\begin{array}{c} L-1\\ I-1 \end{array}\right)$ kinds of probe placements can be selected after locking the initial probe. In an attempt to reconstruct the frequency with high probability, minimal μ(Q) under proper probe placement need to be calculated.

It can be concluded from the simulation results that several arrangements of probe placements, which satisfies the minimal two-sided coherence of matrix **Q**, can be chosen in BTT measurement. Assuming that *L* is set as a constant 25, the relationship between the two-sided coherence of **Q** and the number of probes is shown in Table 1.

Table 1 shows that increasing the number of probes can in some extent improve the incoherence of matrix **Q**. However, (1+1/μ(Q))/2 is not more than $\frac{1}{2}\times \left(1+1/0.4\right)=1.75$, which seems that spectra containing multi-mode frequencies can hard to be recovered. Fortunately, pioneers [25] have proved that the sparse solution still has high probability to be recovered with a μ(Q) exceeding the bounded value.

In summary, optimized probe placements can be selected through finding the minimal μ(Q). It must be noted that the uniform placement of probes must be avoided because μ(Q) in that case is larger than any other kinds of placements regardless of the number of probes.

## 4. Quantitative Expression and Reduction of Measurement Uncertainties

### 4.1. Mathematical Model of Uncertainties in the BTT Measurement

Measurement uncertainties are inherently present among the tip timing signals. Those uncertainty factors could cause aliasing in determining the TOA. Two main factors demonstrated in references are system noise [13] and probe position shift [11]. The deviations of blade tip displacement measurement results due to those factors will be represented in mathematic model respectively.

#### 4.1.1. Uncertainty Due to BTT Signal Noise

**Figure 4:**
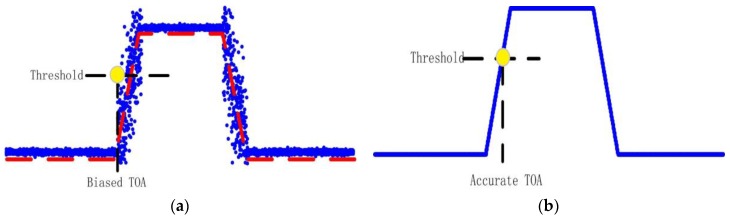
TOA measurement principle (**a**) noise-corrupted case (**b**) noise-free case.

The best raw signal from a BTT probe is similar to a sharp square wave. During the measurement procedure, TOA must be selected from the square wave such as that shown in Figure 4b. TOA is defined as the time at which the signal exceeds a scaled threshold.

However, the existence of BTT signal noise can actually cause difficulty for the timing system to choose consistent and accurate TOA points. As shown in Figure 4a, the system noise can cause rising edge start detection algorithms to be inconsistent. Moreover, noise added to the front face of the BTT signal could cause the optical fiber probe triggered at the incorrect time.

In general, signal noise is always represented as zero mean white Gaussian noise. These assumptions would lead to the following actual blade arrival time:

(15) $$t_{\mathrm{actual}}=t_{\mathrm{actual}}^{0}+e_{\mathrm{noi}},$$

where $t_{\mathrm{actual}}^{0}$ is the ideal TOA of the blade tip, $e_{\mathrm{noi}}$ is zero mean white Gaussian noise with variance $\sigma_{\mathrm{noi}}$. According to Equation (2), the biased TOA can cause the fluctuation of finite time increments $\Delta t_{0}$. Thus, based on Equation (3), the deflections of blade tips biased by the signal noise are found as follows:

(16) $$y=\frac{2\pi R_{\mathrm{tip}}}{T_{n}}\left(\Delta t_{0}+e_{\mathrm{noi}}\right)=y_{0}+\frac{2\pi R_{\mathrm{tip}}}{T_{n}}e_{\mathrm{noi}},$$

where *y_0_* is the true value of blade tip deflection. Since $\frac{2\pi R_{\mathrm{tip}}}{T_{n}}$ is a constant, the blade tip measurement sequences can be modeled as the ideal results added with white noise sequence.

In practice, the noise level or the maximum measurement uncertainty of the sensor is generally provided in its handbook as an important parameter. So the uncertainty of tip deflection due to BTT signal noise can be estimated in some extent.

#### 4.1.2. Uncertainty Due to Probe Position Shift

**Figure 5:**
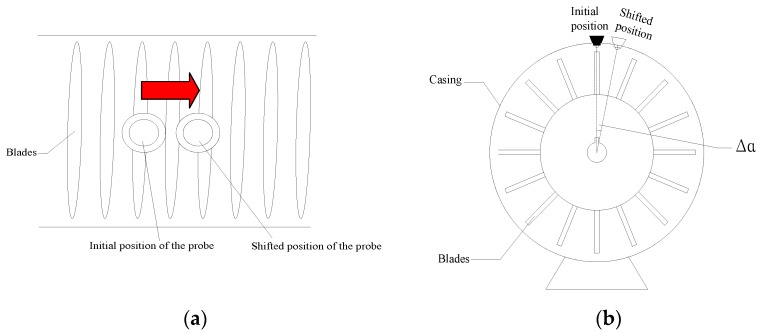
Shift in probe position. (**a**) Top view of blade assembly system (**b**) Front view of blade assembly system.

Reference [11] indicated that expansion of casing is unavoidable due to thermal and mechanical loads. Thus, engine resonance and radial growth of casing can cause the shift of probe position. The shifted positions of optical probes are displayed in Figure 5 from different viewing points.

As can be seen from Figure 5b, the angular position of probes fluctuated under the shift of probe position. The error to original angular position is defined as Δα.

When the position of the sensors are shifted, the time that sensors are expected to be triggered $t_{\mathrm{expected}}$ must be changed simultaneously.

(17) $$t_{\mathrm{expected}}=\frac{T_{n}}{2\pi }\left[\left(\alpha +\Delta \alpha \right)+2\pi n\right]=t_{\mathrm{expected}}^{0}+\frac{T_{n}}{2\pi }\Delta \alpha ,$$

where $t_{\mathrm{expected}}^{0}$ is the expected arrival time without measurement error. As the shift of the probe position has no effect on the accuracy of TOA, the finite time increment is shifted $\frac{T_{n}}{2\pi }\Delta \alpha$ from the ideal one.

According to Equation (3), displacement of blade tip can be found as follows:

(18) $$y=\frac{2\pi }{T_{n}}R_{\mathrm{tip}}\left(\Delta t_{0}+\frac{T_{n}}{2\pi }\Delta \alpha \right)=y_{0}+\Delta \alpha R_{\mathrm{tip}},$$

where $\Delta t_{0}$ is the ideal finite time increment and $y_{0}$ is the ideal blade tip displacement.

Under the irregularly interrupted load, the position shifts of probes Δα in each rotation can be seen as independent random variables. As the long-time continuous testing will be done, measurement results are plentiful. The central limit theorem says that the computed values of the average of Δα will be approximately normally distributed. Reference [11] indicate that true value is expected to lie within $\pm {\left|\Delta \alpha \right|}_{\max}$ no less than 95 percent of time i.e., $P\left\{-{\left|\Delta \alpha \right|}_{\max}\leq \Delta \alpha \leq {\left|\Delta \alpha \right|}_{\max}\right\}=95\%$. In practice, the boundary of angular position can be determined by studying the Finite Element-based data of engine structural [26].

Thus, the inaccuracy level of probe position $\sigma_{\mathrm{pos}}$ can be estimated based on the theory of probability and statistics.

### 4.2. Uncertainties Reduction in Frequency Recovery

Based on Equations (16) and (18), the blade tip displacement considering measurement uncertainties can be re-written as follows:

(19) $$y\left[j\right]=y_{0}\left[j\right]+e_{nos}+\delta_{pos},$$

where $y_{0}\left[j\right]$ is the ideal BTT sampling sequence, It has been proved in the preceding part of the text that $e_{nos}∼N\left(0,\sigma_{\mathrm{nos}}^{2}\right)$ and $\delta_{pos}∼N\left(0,\sigma_{\mathrm{pos}}^{2}\right)$. Variances can be selected from the parameters defining the level of corresponding normal distributions.

According to Equation (9), the measurement sequence can be rewritten in the form:

(20) $$y\left[j\right]=R\theta_{0}\left(f\right)+\delta_{mea},$$

where $\delta_{mea}=e_{nos}+\delta_{pos}$ is the additive error sequence. So the variance of $\delta_{mea}$ can be calculated through root-sum-squaring two uncertainty factors [27].

(21) $$\sigma_{\mathrm{mea}}^{2}=\sigma_{\mathrm{noe}}^{2}+\sigma_{\mathrm{pos}}^{2}.$$

Therefore, the uncertainty level of the total system can be estimated by combining the corrupting level of each uncertainty factor respectively. To make the sparse solution more closely to real frequency spectrum $\theta_{0}\left(f\right)$, Basis Pursuit De-noising (BPDN) method [28] is applied to this problem.

First, the reconstruction problem can be transformed based on Equation (10) when measurement results are corrupted.

(22) $$Ty\left[j\right]=Q\theta_{0}\left(f\right)+T\delta_{mea},$$

where $T=QR^{†}$ has been defined as the transformation matrix.

BPDN method adapts to de-noised spectrum $\hat{\theta }\left(f\right)$ through the solution of the convex unconstrained optimization problem:

(23) $$\underset{\hat{\theta }\left(f\right)}{\min}\;\frac{1}{2}{\left\|z-Q\hat{\theta }\left(f\right)\right\|}_{2}^{2}+\lambda {\left\|\hat{\theta }\left(f\right)\right\|}_{1},$$

where **z** = **Ty**[*j*] is the transformed measurement sequence. The key step of BPDN involves finding a quadratically constrained linear program that is closely related to the following convex constrained optimization problems [29]:

(24) $$\underset{\hat{\theta }}{\min}\;{\left\|\hat{\theta }\left(f\right)\right\|}_{1}\;s.t.\;{\left\|z-Q\hat{\theta }\left(f\right)\right\|}_{2}\leq \varepsilon ,$$

where ε is nonnegative real parameters. If the error term of in Equation (24) satisfies ${\left\|T\delta_{mea}\right\|}_{2}\leq \varepsilon$, then the solution will be close to $\theta_{0}\left(f\right)$ with high probability. Therefore, it can be concluded that as far as the level of uncertainty factors is estimated accurately, efficient algorithms [29] have been proposed for obtaining $\hat{\theta }\left(f\right)$ which close to the true value.

In practice, a feasible way for estimation of ε is based on the estimation of $\delta_{mea}$. Here the experimental biases are conducted to find out the uncertainty parameter $\sigma_{\mathrm{mea}}$ in the total measurement chain. Therefore the error sequence $\delta_{mea}$ can be regarded as a standard white Gaussian noise with a noise level $\sigma_{\mathrm{mea}}$. Finally the boundary of ε can be determined by calculating ${\left\|T\delta_{mea}\right\|}_{2}$.

## 5. Numerical Simulations

### 5.1. Recovery of Multi-Mode Signals

This section a sequence of artificial multi-mode vibration signals is presented to validate the ability of BP and BPDN method in detecting the multiple harmonic frequencies. Based on Equation (5), the simulated signal is designed as follows:

(25) $$d\left(t\right)=\sum_{i=1}^{3}A_{i}\sin\left(2\pi f_{i}t+\varphi_{i}\right),$$

where $f_{i}=\left\{332,482.2,599.3\right\}$, which contains one synchronous mode and two asynchronous modes, amplitudes are set as $A_{i}=\left\{0.3,0.2,0.1\right\}$, $\varphi_{i}\in \left[0,2\pi \right]$ are the randomly selected parameters.

**Figure 6:**
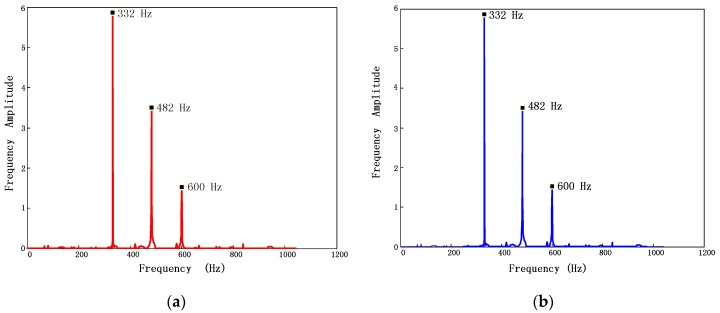
Comparison of frequency recovery of unbiased BTT data (**a**) Frequency spectrum recovered by BP (**b**) Frequency spectrum recovered by BPDN.

The BP method is firstly adopted in the simulated signals and the parameters of BP are set as: the parameter of rotating speed *f_r_* in simulation model is fixed as 5000 *r/min* (83.3 Hz); the parameters of probe number are selected to satisfy the uniqueness of solution and thus *L* = 25, *I* = 4; the parameter of probe positions Λ={1,3,8,19}; *N* is the number of rotation in testing which should be long enough to get the abundant information and thus *N* = 70. Therefore, the frequency 332 Hz is the synchronous mode whose engine order (EO) equals to four. All the simulated BTT signals are sampled using the SIMULINK function Hit Cross [30]. The reconstructed frequency spectrum of the vibration signal is displayed in Figure 6a.

BPDN method is then introduced to detect the harmonic features of vibration signals. To compare with BP method, all the parameters in BPDN method are the same as those in BP. In addition, the parameter of noise level ε is set as zero because the simulated BTT signals are uncontaminated.

The results of the extracted harmonic frequencies are shown in Figure 6b. Comparing the two proposed methods, it can be verified that both methods have well performance in detecting the harmonic frequencies of vibration signals without biased signals. Furthermore, the dominant mode of vibration can be clearly identified through the amplitudes of reconstructed spectrum.

### 5.2. Reduction of Measurement Uncertainties

In this section, simulation experiments are designed to illustrate the ability of the proposed method in reducing effects of uncertainties in spectra recovery. The simulation signal can be designed as a double-mode signal corrupted by measurement uncertainties.

(26) $$d\left(t\right)=A_{1}\sin\left(2\pi f_{1}t\right)+A_{2}\sin\left(2\pi f_{2}t\right)+v\left(t\right).$$

According to Equation (21), v(t) can be formulated as a zero-mean normally distributed signal comprised of two major uncertainty factors (noise and position shift). Hence various measurement uncertainties has been converted into a single additive signal sequence.

To describe the influence of measurement uncertainties and the accuracy of frequency recovery, noise-to-signal ratio(NSR) [31] and probability of recovery(*β*) [32] are introduced respectively, defined as:

(27) $$NSR=1/20\log\left(\frac{{\left\|d\left(t\right)-v(t)\right\|}_{2}}{{\left\|v\left(t\right)\right\|}_{2}}\right),$$

(28) $$\beta =1-{\left\|\theta \left(f\right)-\hat{\theta }\left(f\right)\right\|}_{2}/{\left\|\theta \left(f\right)\right\|}_{2}.$$

**Figure 7:**
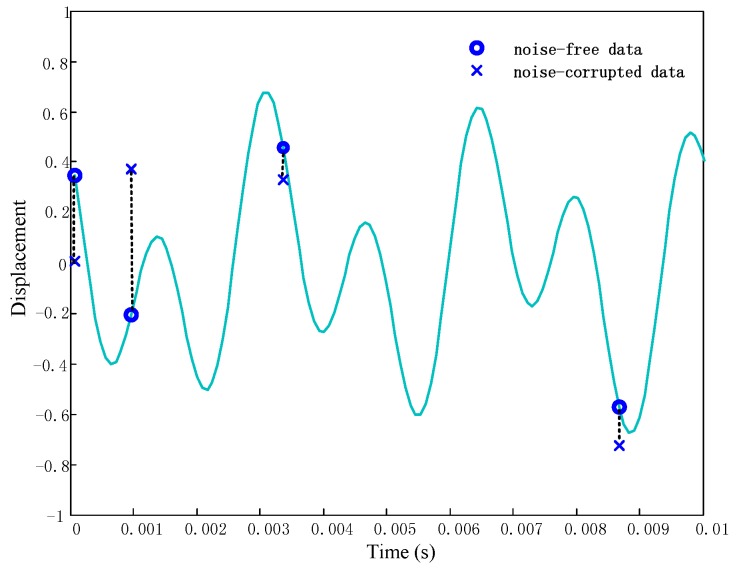
Ideal BTT signals and corrupted signals sampled by four probes in one rotation.

Firstly, a specific simulated signal is applied to BP and BPDN method respectively. The parameters of testing signal are set as: *A_1_* = 0.4, *A_2_* = 0.3, *f_1_* = 600 Hz, *f_2_* = 280 Hz, *NSR* = 0.3. Parameters in measurement system are the same as those in Section 5.1. The waveform and sampled data of simulated signals are presented in Figure 7.

**Figure 8:**
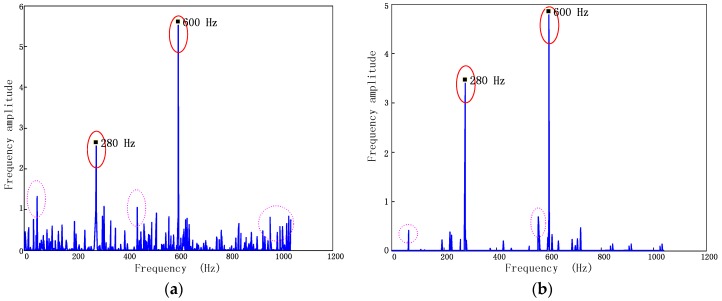
Comparison of frequency recovery of biased BTT data (**a**) Frequency spectrum recovered by BP(*β* = 31.29%, $\frac{A_{1}}{A_{2}}\approx 2\text{:}1$) (**b**) Frequency spectrum recovered by BPDN(*β* = 56.03%, $\frac{A_{1}}{A_{2}}\approx 4\text{:}3$).

Figure 8a shows the recovered frequency spectrum by BP method. It can be seen that the noise has masked the dominant harmonic components. Considering the uncertainty level by importing the parameter ${\left\|Tv\right\|}_{2}\leq \varepsilon$, frequency spectrum recovered through BPDN method is shown in Figure 8b. Obviously, the noise has been eliminated and the dominant frequencies can be observed more clearly.

Then, comparison of both methods was performed using Monte Carlo simulations.

The parameters of simulated signals are set as follows: amplitudes A_1_ and A_2_ are selected randomly in each test from the range (0, 1]. While the frequency *f_1_* and *f_2_* are selected randomly from (100, 1000] Hz.

**Figure 9:**
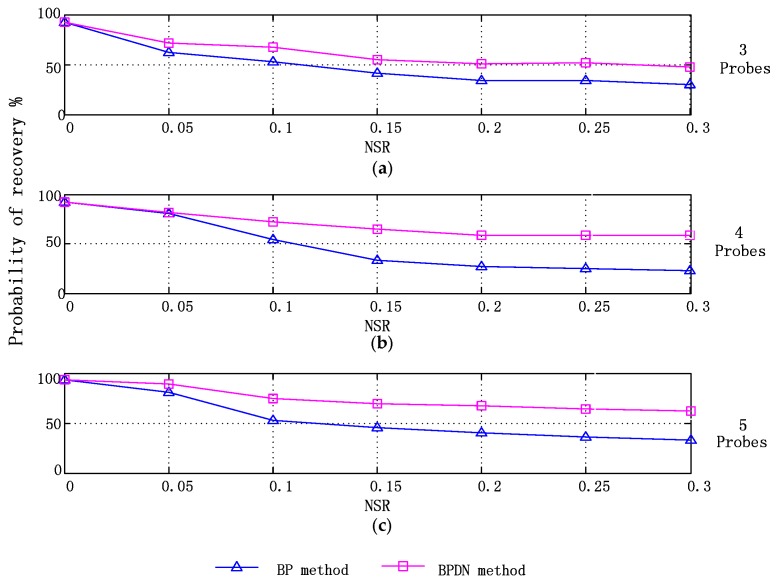
Reconstruction accuracy of BP and BPDN methods using (**a**) 3 probes (**b**) 4 probes (**c**) 5 probes.

A number of probes ranging from three to five are used for sampling and recovering vibration signal. For each probe number, a set of angular position of probes which satisfies the minimal *μ*(**Q**) are selected and fixed during the simulation experiment. Six NSR values are selected from 0.05 to 0.3. The noises in different level will be added to original signals in test. 100 signals are generated under each NSR for the testing. The average of *β* in 100 experiments are calculated in each case of testing and displayed in Figure 9.

Compared with BP, the BPDN method alleviated to a certain extent the effect of the noise and made an appealing improvement in identifying blade vibration frequencies without increasing the probe number.

## 6. Experiments

Two experiments are performed in this section to validate the ability of the proposed method in multi-frequency detection and measurement uncertainty reduction using respective test rigs.

### 6.1. Recovery of Multi-Mode Vibration Response in Frequency Domain

**Figure 10:**
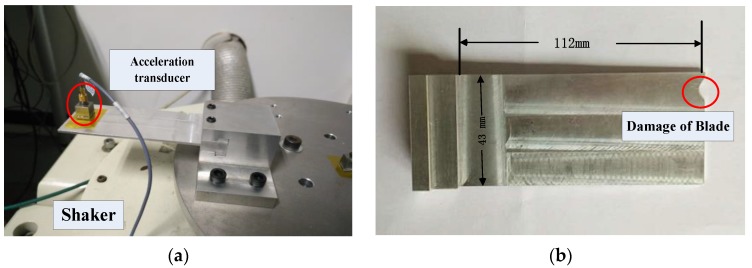
Vibration-based test rig (**a**) photo of testing rig (**b**) damaged testing blade of AL7075.

Rotor blades often generate multi-mode vibration response in working conditions when suffered from damage. Therefore, a vibration-based test is performed to acquire the multi-mode tip deflection signals. The vibration-based test is run using the DV-100-1 (980N) electrodynamic shaker and the rig is shown in Figure 10. The test specimen is designed according to the size and material of a rotor blade for a third stage of a compressor. To match the working case more exactly, a cut is made on the blade tip and the testing blade is attached to the fixture with dovetail joints, which is the same with the root attachment of compressor disk. The tip displacement signals are measured by the acceleration transducer on the front of the blade under the sampling rate 24,000 Hz.

**Figure 11:**
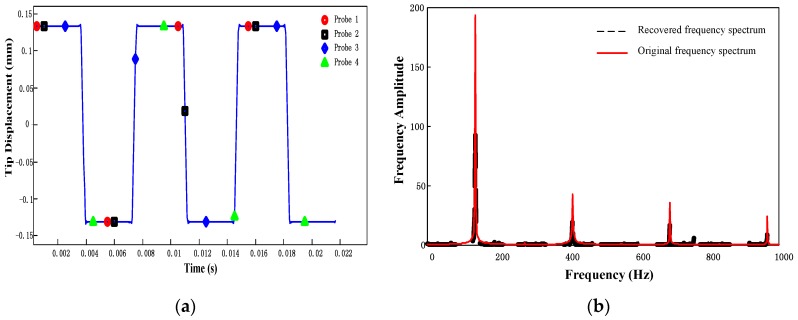
Tip vibration signals and its corresponding spectrums (**a**) tip deflection data sampled by acceleration transducer and BTT sampling system (**b**) frequency spectrum of original vibration signal and recovered spectrum by BP method.

The experiment is implemented at the excitation frequency of 136 Hz, which is close to the resonant frequency of the testing blade. Under the fixed excitation load, the vibration signal and its frequency spectrum are shown in Figure 11. It can be found that the response of blade tip vibration under a single harmonic excitation consists of multiple harmonic signals. Thus, the recovery of multi-mode signals in frequency domain has been proved to be meaningful.

Although the BTT signals of the compressor cannot be acquired directly, a sub-sampled sequence can be extracted from the continuous tip vibration signals to represent the BTT sampling sequence equivalently. Suppose the testing blade is attached to a disk and rotated under the speed *f_r_* = 200 Hz. Probe numbers are selected to satisfy the uniqueness of solution and thus *L* = 20, *I* = 4. The positions of probes Λ={1,3,9,17} are selected by calculating minimal two-sided coherence. According to the sampling time of the imaginary BTT measurement above, equivalent BTT signals can be manually extracted from the continuous vibration signals sampled by the acceleration sensor as shown in Figure 10a. BP method is then introduced to recover the vibration frequency spectrum using the parameters mentioned above.

The recovered frequency spectrum is shown in Figure 11b. Comparison of recovered frequency spectrum with the original one confirms that the proposed method is effective in detection of multi-mode frequency in the engineering applications.

### 6.2. Uncertainty Reduction in Frequency Detection Results

**Figure 12:**
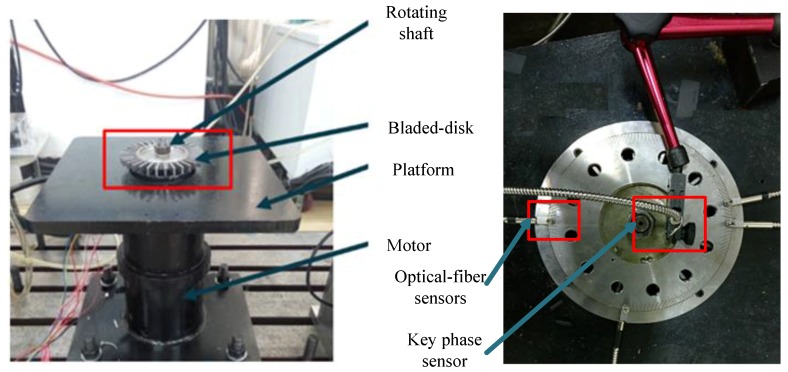
The test rig.

The rotating blades often generate vibration at certain speed. Thus, the BTT measurement is performed under a single engine-ordered excitation. The experimental test rig is shown in Figure 12. Four optical-fiber probes are embedded in the circular case to sample arrival times. The key phase measurement probe is placed close to the rotating shaft for sampling reference time impulses. The system is driven by a motor whose speed could reach 8000 RPM. The experiment is performed through 32 blades made of corrosion-resistant chromium steel with overall sizes of 34 mm long, 11 mm wide and 3 mm thick.

The validation experiment is performed as follows. First, the Campbell diagram of rotating blades is completed based on the finite element (FE) analysis results and the EO excitation speed *f_r_* is selected according to the diagram. Then the test is implemented at the certain excited speed *f_r_*. Finally, the time impulse signals of the blades are sampled to calculate the tip deflection. Considering the fluctuation of the rotation system, a continuous testing is done to select data sampled undergoing a stable rotating speed.

**Figure 13:**
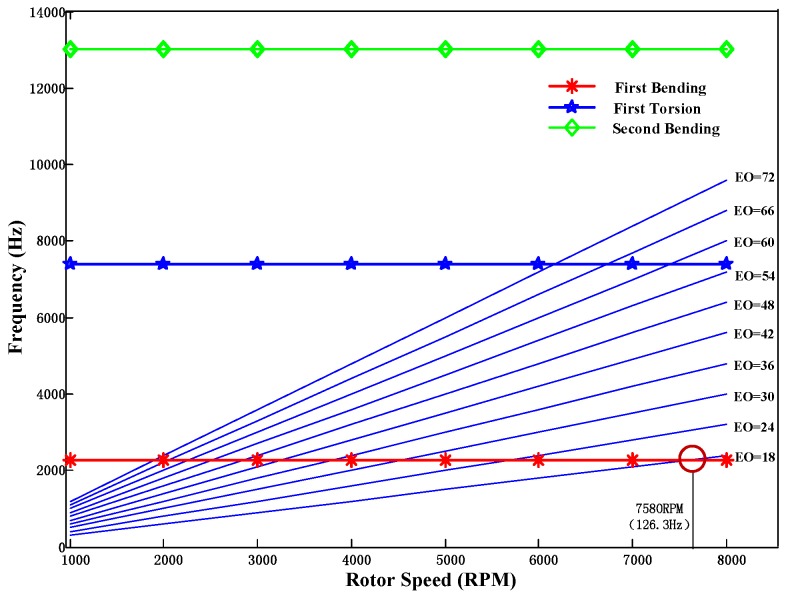
Campbell diagram of experimental setting blade first three modes with 18 EO crossing at 2278 Hz and 7580 rpm.

**Figure 14:**
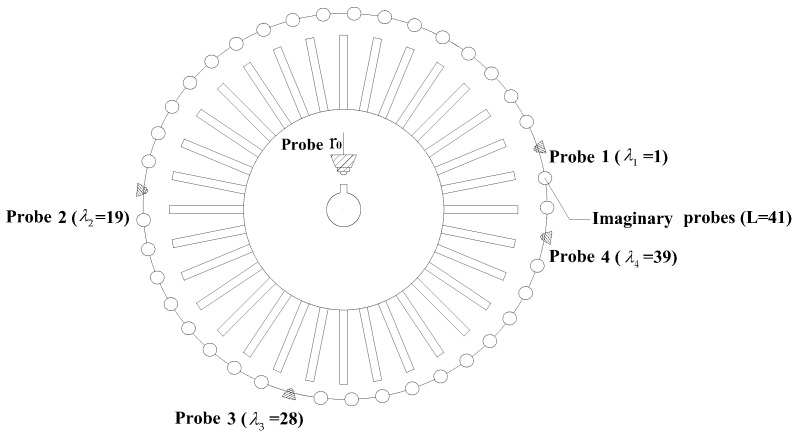
Placement of optical probes.

The Campbell diagram of the rotating blade is shown in Figure 13 from which the EO excitation speed can be adjusted according to the point where the first bending frequency curve crosses one of the EO excitation lines. It has been proved that the blade will be excited if the parameters of EO excitation are set as EO = 18, *f_r_* = 126.3 Hz [33]. Here the parameter of BP method *L* is initialized as 41 according to the results for the first bending modes in FE model. Based on the analysis in Section 3.2.2, a probe placement method is decided as *Λ* = {1, 19, 28, 39} and shown in Figure 14.

**Figure 15:**
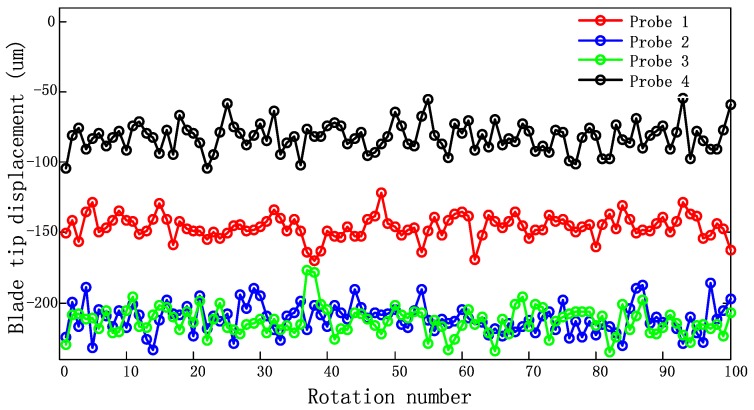
Signals of blade tip displacement.

Vibration signals of blade 18 are shown in Figure 15. The tip deflections collected by four probes are fluctuating around the same phrase respectively, which shows the blade resonate at a synchronous vibration and the sampling signals are corrupted.

**Figure 16:**
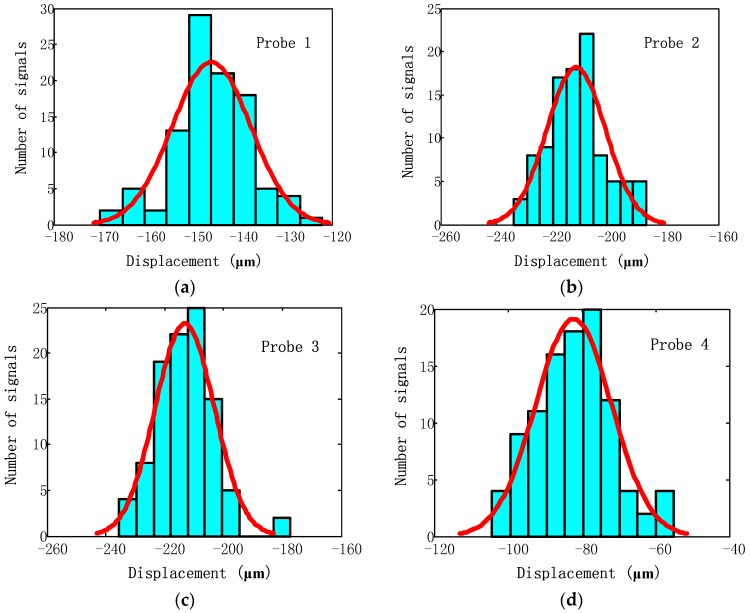
Distribution of signal sampled by (**a**) Probe 1 (**b**) Probe 2 (**c**) Probe 3 (**d**) Probe 4.

The distribution of measurement results of four probes is shown in Figure 16. It can be seen that displacements that are detected by each probe approximately obey the normal distribution. Hence, it can be concluded that the uncertainty measurement model built above is feasible. Furthermore, the uncertainty level of the measurement $\sigma_{\mathrm{mea}}$ can be calculated through the distribution in Figure 16. As the optical probes are fixed to the platform in this experimental rig, the source of jitter for about 20 μm is mainly caused by intrusion of optical noise and the limitation of sensor resolution rather than shift of probe position.

**Figure 17:**
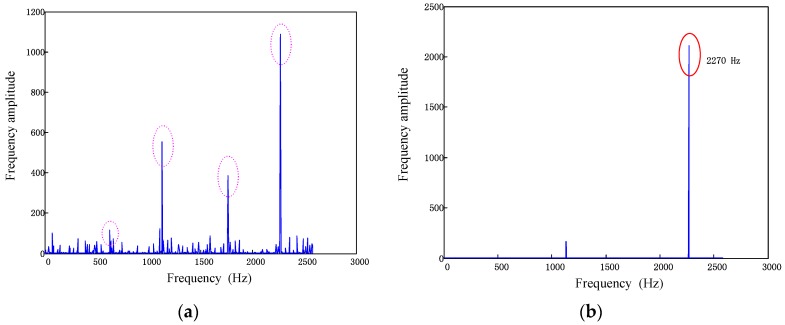
The recovered spectrum of sample streams based on (**a**) BP algorithm (**b**) BPDN algorithm.

With the sampled signals, a single-measurement vector model can be built and the frequency spectrum can be recovered by BP algorithm as Figure 17a. Then the parameter ε is calculated and the frequency spectrum is recovered based on BPDN method. The result of the advanced method is shown in Figure 17b.

By comparing Figure 17a with Figure 17b, it can be seen that the spectrum reconstructed by the BP algorithm is mixed with some biased frequency characteristics while the spectrum reconstructed with the de-noise method is clean and the dominant frequency can be extracted easily. Furthermore, the frequency detected by the BPDN method was well accorded with the Campbell diagram.

It must be noted that due to high stiffness of the material and the limited excitation method in our current equipment, only a single synchronous mode of the blades can be excited. Our further work might focus on acquiring and analyzing BTT sampling data that contains multi-mode vibration components and measurement uncertainty simultaneously from compressors or jet engines to validate the proposed method.

## 7. Conclusions

BTT-based monitoring of the vibration characteristics of rotating engine blades is crucial for fault diagnoses. However, two of its main drawbacks are that BTT signals are under-sampled and tend to be biased under the uncertainty factors in measurement. This paper explores a novel BTT analysis way for alleviating uncertainties in recovery of multi-mode blade vibration signals based on sparse theorem. With the probe number not more than those used in conventional methods, recovering four or even more frequency components from the blade vibration signals can be available. Moreover, without increasing the probe number, the probability of frequency recovery can be maintained higher than 50% under a certain range of noise level. Simulation and experimental results confirm that the proposed method dramatically improved the capacity of monitoring vibration characters of compressor blades, especially when the dynamic behavior of rotated blades is highly nonlinear.

The main results are summarized as follows:

1. A single-measurement vector model for sparse BTT data is built and all the harmonic frequencies can be extracted from the solution of the sparse representation model if the measurement results are unbiased.
2. The number and placement of optical-fiber probes play an important role in the effectiveness of frequency spectrum recovery. Considering the blade vibration conditions in practice, four probes located around the case, which satisfies the minimal two-sided coherence, is enough to recover the vibration frequency spectra well.
3. The improved blade vibration recovery method based on BPDN obviously enhanced the robustness to the noise interferences. The dominant vibration frequencies can be identified from corrupted non-uniformly under-sampled BTT data through the proposed method without increasing the number of probes.

## Figures and Tables

**Table 1:** Minimal two-sided coherence of matrix **Q** (*L* = 25).

Number of Probes *I*	Minimal μ(**Q**)
2	1
3	0.893
4	0.691
5	0.500
6	0.478
